# Supplementary figures and images for: Nutraceutical Profile Characterization in Apricot (Prunus armeniaca L.) Fruits
Source: Plants (Basel). 2025 Mar 22;14(7):1000. doi: 10.3390/plants14071000 (PMC11990447; doi:10.3390/plants14071000)

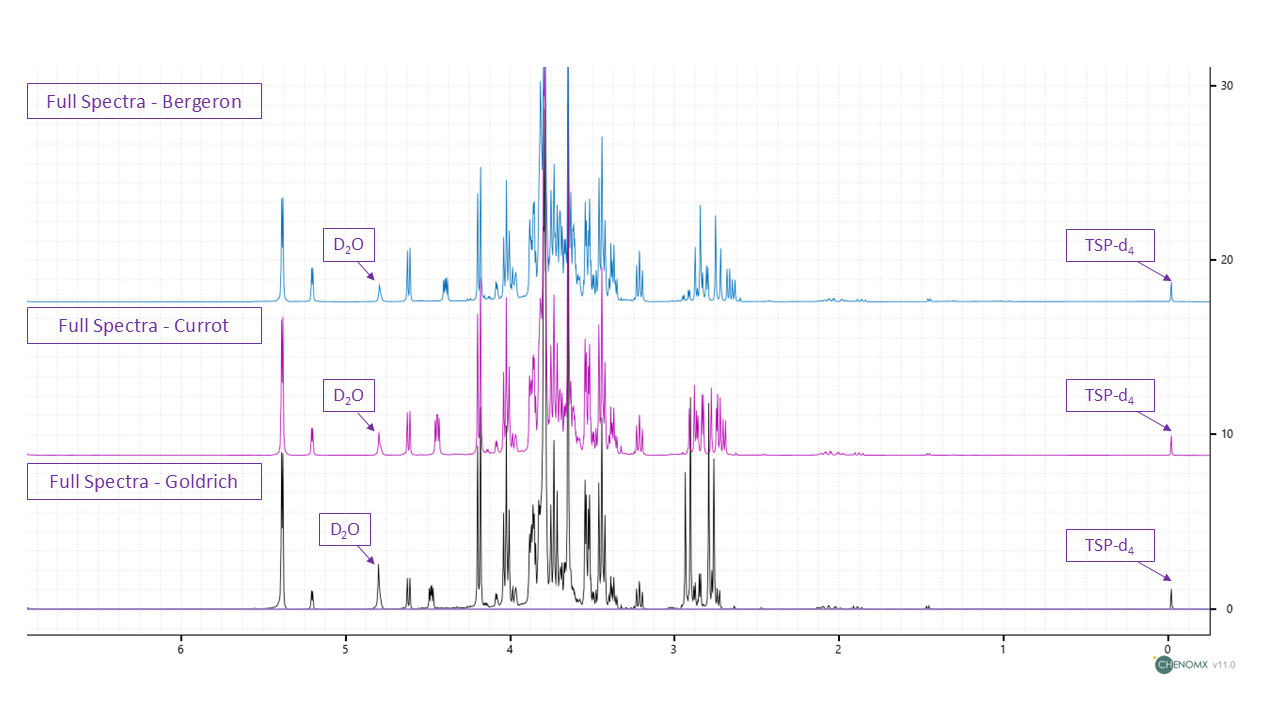

Supplement: Supplementary file 1 [file plants-14-01000-s001.zip › Figure S1.png]
